# The Influence of the External Chemistry of Silica-Based Mesoporous Nanocarriers on Organ Tropism and the Inhibition of Pulmonary Metastases

**DOI:** 10.3390/pharmaceutics17111389

**Published:** 2025-10-26

**Authors:** Wenping Ye, Yakai Yan, Liuyi Chen, Zhongrui Yang, Guangya Xiang, Yao Lu

**Affiliations:** 1 School of Pharmacy, Tongji Medical College, Huazhong University of Science and Technology, Wuhan 430030, China; 19828214385@163.com (W.Y.); d202381887@hust.edu.cn (Y.Y.); m202375825@hust.edu.cn (L.C.); 15908643215@163.com (Z.Y.); youjti@mails.tjmu.edu.cn (G.X.); 2 NMPA Key Laboratory for Quality Research and Control of Drug Products, Wuhan Institute for Drug and Medical Device Control, Wuhan 430075, China

**Keywords:** HSMONs, lung tropism, protein corona, albumin, gambogic acid

## Abstract

**Background:** Mesoporous silica nanoparticles (MS NPs) have attracted significant interest for their role in the advancement of drug delivery systems. However, further investigation is needed to unravel the mechanisms behind the shift in organ tropism that occurs with changes in composition. **Methods:** To shed light on the correlation between their composition and organ-targeting capabilities, a range of MS NPs was synthesized and subsequently administered intravenously to mice. **Results:** Our results indicate that MS NPs with a pristine -Si-O-Si- framework, or those incorporating -C-C- or –S-S-S-S- bonds, predominantly accumulated in the liver. The shift to lung tropism was observed exclusively in MS NPs that were enriched with –SH groups. Proteomic analysis identified histidine-rich glycoprotein (HRG) as the most prevalent protein associated with liver-preferred MS NPs in serum, while lung-preferred MS NPs, such as thioether-bridged deformable hollow mesoporous organosilica nanoparticles (HSMONs), showed the highest affinity for albumin. Furthermore, the lung-selective HSMONs, endowed with inherent deformability and glutathione-responsive biodegradability, were utilized as systemic nanocarriers for the delivery of gambogic acid (GA). **Conclusions:** Leveraging albumin absorbing-triggered tumor cell targeting and trafficking, HSMONs conjugated with GA effectively elicited potent antitumor effects in pulmonary tissue.

## 1. Introduction

The approval of COVID-19 mRNA vaccines delivered by lipid nanoparticles (LNPs) has underscored the increasing nanoparticle-based delivery systems [1,2,3,4]. However, after systemic administration, the majority of these nanoparticles are mainly distributed to the liver and spleen, which restricts their application in specific organ targeting. Beyond LNPs, mesoporous silica nanoparticles (MS NPs), featuring tunable mesoporous sizes, high drug loading efficiency, and large surface areas [5,6,7,8,9], have earned a great deal of attention in the fields of drug delivery, disease diagnosis, theranostics, biosensing, and even tissue engineering due to their unique properties. From numerous biodistribution studies on MSNs, it is evident that most of them are internalized by the reticuloendothelial system (RES) and quickly end up in the liver. Since targeted nanoparticle-based drug delivery can minimize the side effects of conventional pharmaceutical agents and enhance their effectiveness, the fabrication of MS NPs for specific organ targeting has attracted a lot of attention [10,11,12]. Size, geometry, porosity, and surface characteristics might have an impact on the ultimate in vivo destination [13]. Ghandehari et al. found that increasing the aspect ratio of amine-modified MS NPs from 1 to 8 led to an increased accumulation in the lung [4]. For active targeting, researchers conjugated the ligands/receptors on the surface of MS NPs to achieve targeted delivery [14,15,16].

In addition, following intravenous injection, the outer surface of nanoparticles can rapidly absorb protein corona [17,18]. The formation of the serum protein corona, the role of particle size and surface chemistry in corona formation, and the consequences for in vitro and in vivo behavior have been extensively investigated [19,20]. Some researchers have observed the different protein corona absorbed on the MS NPs after different surface modification in vitro [21]. It has been found that a greater number of proteins, especially apolipoproteins, associate with the MSNs at higher serum concentrations, irrespective of surface chemistry and morphological differences [22,23].

However, detailed studies on biodistribution, efficient particle targeting and particle elimination remains necessary. Moreover, a profound investigation into the correlation between the composition of MS NPs, the protein corona, and in vivo distribution is required. To address the aforementioned issues, we synthesized diverse types of MS NPs and analyzed the correlation between the composition of the MS NPs and the direct outcomes of biodistribution.

We accidentally found the unique thioether-bridged (-S-S-S-S-) deformable hollow mesoporous organosilica nanoparticles (HSMONs) exhibited a preference for accumulating in lung tissue following systemic administration. It was subsequently demonstrated that the -SH group, rather than the tetrasulfide bonds, played a key role in the lung tropism, at least for MS NPs. Conversely, MS NPs lack of -SH groups displayed a preference for liver tissue.

**Scheme 1:**
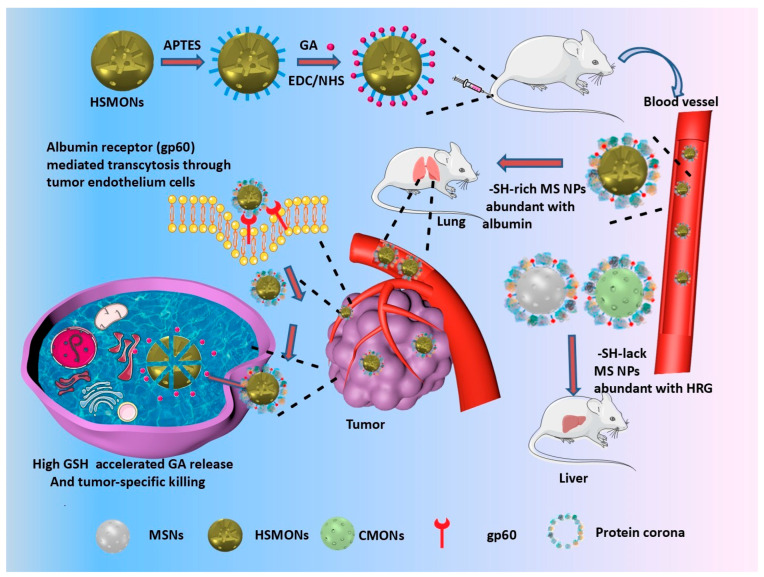
Schematic illustration of the mechanisms of HSMONs for lung tropism and tumor killing.

Since all of the MS NPs we synthesized displayed a positive surface charge due to identical surface modification procedures. In serum, these nanoparticles consistently measured within the nanoscale range, and importantly, none of them were conjugated with targeting ligands. To elucidate the underlying mechanism of HSMONs’ pulmonary tropism, proteomic analysis was employed to identify the protein corona absorbed onto each type of MS NP. Data showed that the liver-selective nanoparticles absorbed the highest amount of histidine-rich glycoprotein; whereas, the lung-selective nanoparticles, such as SMONs and HSMONs, absorbed the highest amount of albumin. Moreover, a higher enrichment of albumin corresponded to greater accumulation of MS NPs in the lungs. Therefore, for MS NPs, a specific kind of nanoparticles, the unique absorption of the protein corona appears to dictate their ultimate in vivo distribution (Scheme 1).

The choice of deformable HSMONs was driven by their highest sulfur content among the lung-selective nanoparticles, and their hollow structure, which further increases the drug loading efficiency. In addition, we speculated that the albumin-absorbing properties and the intrinsic -S-S-S-S- bounds within the framework confer several advantages to HSMONs: 1. lung tropism, which is crucial for targeted drug delivery to the lungs; 2. binding to gp60 albumin receptors and across the lung endothelial cells via transcytosis [24,25,26,27]; 3. the deformable structure of HSMONs enhanced the cellular uptake via a spherical-to-oval morphology change [5,8,28]; and 4. when targeting the tumor site, HSMONs were endocytosed by tumor cells through another albumin receptor-secreted protein acidic and rich in cysteine (SPARC) and the high GSH level triggered the degradation of HSMONs, thereby releasing the payload only inside the tumor cells [29,30].

Given the reasons previously discussed, the lung-preferred HSMONs were employed as the systemic nanocarrier for the delivery the herbal-derived drug gambogic acid (GA), and its anti-tumor efficacy was subsequently assessed. GA, as the main active ingredient in gamboges, has exhibited incredible anticancer activity against various cancer cells with minimal toxicity to normal cells [31]. Its ability to upregulate the expression of p53 and src-3, and downregulate of VEGF, AKT, and NF-κB makes GA a promising candidate in cancer therapy [32,33]. However, its clinical application is hindered by its low aqueous solubility (<5 ppm) and short half-life in the blood stream (t_1/2_ = 15.7 min) [34]. It is therefore logical to conjugate GA to deformable HSMONs for better bioavailability and enhanced antitumor efficacy. Notably, the results were in line with our expectations: the lung-selective HSMONs-GA exhibited superior anti-tumor metastasis efficacy in the lungs compared to liver-selective MS NPs and free GA in the pulmonary.

This study provides proof of concept that by tuning the composition of the MS NPs, we can achieve specific lung-targeting capabilities through the absorption of a featured protein corona. This finding may offer valuable insights into the relationship between the composition of NPs and their bio-destination, at least within the context of mesoporous silica nanoparticles.

## 2. Materials and Methods

### 2.1. Materials

Bis(triethoxysilyl) ethane (BTEE), and 3-aminopropyltriethoxysilane (APTES) were ordered from RHAWN (Shanghai, China). Cetyltrimethylammonium chloride (CTAC), anhydrous ethanol, hydrochloric acid (37%, *w*/*w*), concentrated ammonia aqueous solution (25–28 wt%), 4-dioxane, NaH_2_PO_4_, Na_2_HPO_4_, and dimethyl sulfoxide (DMSO) were obtained from Sinopharm Chemical Reagent Co, Ltd. (Shanghai, China). Tetraethyl orthosilicate (TEOS), sodium hydroxide (NaOH), triphenylphosphine, glutathione (GSH), 1-ethyl-3-[3-dimethylaminopropyl] carbodiimide hydrochloride (EDC), and N-hydroxy-succinimide (NHS) were obtained from Aladdin Industrial Inc. (Shanghai, China). Bis [3-(triethoxysilyl) propyl] tetrasulfide (BTES) and 5,5′-dithio bis-(2-nitrobenzoic acid) (DTNB) were purchased from Sigma-Aldrich Chemical Co (St. Louis, MO, USA). Gambogic acid (GA) (HPLC > 98%) was purchased from Weikeqi Bio-Tech Co, Ltd. (Chengdu, China). Sulfo-Cyanine7 Succinimidyl Ester (Cy7 SE) and Sulfo-Cyanine3 Succinimidyl Ester (Cy3 SE) were purchased from MedChemExpress. Roswell Park Memorial Institute 1640 medium (RPMI 1640) and fetal bovine serum (FBS) were purchased from Gibco (Thermo Fisher Scientific, San Francisco, CA, USA). All chemicals were used without further purification. Deionized water was used in all synthetic experiments.

### 2.2. Synthesis of MS NPs

Mesoporous silica nanoparticles (MS NPs) were prepared first according to the previous reports as the following methods [5,8]. Firstly, cetyltrimethylammonium chloride (CTAC, 600 mg) was dissolved in a mixture containing deionized water (225 mL), ethanol (90 mL), and concentrated ammonia aqueous solution (3.0 mL, 25–28 wt%). This mixture was then heated at 35 °C in an oil bath and followed by adding 1.05 mL tetraethyl orthosilicate (TEOS) under vigorous stirring at 1100 rpm. The mixture was stirred at 35 °C for 24 h to allow for the formation of CTAC@MSNs. These were then collected by centrifugation, washed with ethanol three times, and dried under a vacuum. The CTAC templates were then removed from the MSNs by solvent extractions thrice in a solution containing concentrated HCl (240 μL, 37% *w*/*w*) and ethanol (120 mL) at 60 °C for 3 h. After washing with ethanol three times, the MS NPs were obtained through vacuum drying.

The synthetic procedure for mesoporous organosilica nanoparticles (CMONs), and dried thioether-bridged periodic mesoporous organosilica nanoparticles (dried-SMONs) followed a similar process to that of MSNs except for the replacement of TEOS with corresponding multiple-precursor mixtures. In the case of CMONs, the silica source precursors were used in the following volumes: 0.3 mL of BTEE and 0.75 mL of TEOS. For dried-SMONs, the silica source precursors were adjusted to 0.3 mL of BTES and 0.75 mL of TEOS. For SMONs-calcination synthesis, the prepared dried-SMONs were calcinated at 450 °C for 4 h to remove organic components.

The preparation of thioether-bridged hollow periodic mesoporous organosilica nanoparticles (HSMONs) is described as follows: The 350 mg CTAC@SMONs, after drying, were etched with NaOH (0.16 M) at room temperature. After 30 min, the product was collected by washing three times with a mixture of ethanol and deionized water. The CTAC template was removed from the HSMONs through solvent extraction at 60 °C three times for 3 h, and then the HSMONs were finally obtained by vacuum drying.

Non-dried-SMONs were prepared with 0.3 mL BTES and 0.75 mL TEOS precursors. After vigorous stirring at 35 °C for 24 h, the prepared CTAC@SMONs were collected in a centrifuge tube and washed three times with ethanol. Vacuum drying is not necessary for this step. The CTAC templates were then removed from the organosilica by three solvent extractions in a solution containing concentrated HCl (240 μL) and ethanol (120 mL) at 60 °C for 3 h. After washing with ethanol three times, the non-dried-SMONs were obtained by vacuum drying. For the preparation of non-dried-SMONs-SH, 30 mg of non-dried-SMONs were dispersed in a mixed solution containing 1.1 mL of dioxane, 0.3 mL of water, and 0.10 g of triphenylphosphine. The resulting solution was then heated to 40 °C, and two drops of concentrated aqueous HCl solution (37% *w*/*w*) were added. After being maintained under a nitrogen atmosphere for 2 h, the disulfide bonds in the non-dried-SMONs were reduced to thiol groups. After the obtained thiol group incorporated non-dried-SMONs were washed with ethanol three times, and the non-dried-SMONs-SH were finally obtained through vacuum drying.

### 2.3. Functionalization of MS NPs with Amine Groups of APTES

The surface of the MS NPs was functionalized with amine groups through treatment with APTES. In a typical procedure, the as-prepared MS NPs (20 mg) were re-dispersed in 40 mL of ethanol and subjected to sonication. Following this, the solution was stirred at 78 °C for 30 min to ensure thorough mixing. Then, 20 μL of APTES was added to the mixture, which was subsequently refluxed at 78 °C for 24 h to complete the surface functionalization. After the reaction, the amine-modified MS NPs (MS NPs-NH_2_) were washed three times with ethanol to remove any unreacted APTES.

### 2.4. Synthesis of Gambogic Acid @MS NPs

To conjugate the gambogic acid (GA) on the surface of MS NPs-NH_2_, 7.2 mg MS NPs-NH_2_ was dispersed in 0.73 mL of DMSO. Then, the suspension of MS NPs-NH_2_ was mixed with 2 mg free GA, 6.32 mg EDC and 0.76 mg NHS. The pH of the solution was adjusted to 8.2 to optimize the conjugation conditions. The resulting suspension was stirred overnight (12 h) at room temperature in the dark. Following the GA conjugation, the modified nanoparticles were isolated by centrifugation for 15 min and washed five times with DMSO to remove any unconjugated GA. Determination of GA Conjugation Efficiency: GA@NPs were prepared according to the above method, and the supernatant was collected. Three replicate wells were set for measurement. The absorbance was measured using a microplate reader at the maximum absorption wavelength of 360 nm. The GA conjugation efficiency (%) was calculated as (W_t_ − W_F_)/W_t_ × 100%. The drug loading efficiency (%) was calculated as (W_t_ − W_F_)/(W_t_ − W_F_ + WNP) × 100%, where W_t_ is the total weight of the drug added, W_F_ is the weight of the unbound (free) drug, and WNP is the total weight of the drug-loaded nanoparticles.

### 2.5. Synthesis of Cy7 SE/Cy3 SE@MS NPs

Typically, 1 mg MS NPs-NH_2_ was ultrasonically dispersed in 0.99 mL DMSO, 10 μL Cy7 SE or Cy3 SE (1 mg/mL DMSO) was added. The mixture was allowed to react at room temperature in a dark environment for a period of 2 h, resulting in the formation of Cy7 SE (or Cy3 SE) conjugated MS NPs. The Cy7 SE/Cy3 SE analysis were similar to GA. The measurements were performed using a microplate reader at a wavelength of 750/550 nm.

### 2.6. Characterizations of MS NPs

The internal structure of the samples was evaluated by TEM Field emission (Tecnai G2 20, FEI, Eindhoven, The Netherlands), a transmission electron microscope (FTEM, Tecnai G2 20, FEI, Eindhoven, The Netherlands) and energy-dispersive X-ray spectroscopy. The minute quantities of samples were carefully mounted on the copper grids for characterization. The X-ray photoelectron spectroscopy (XPS) patterns were operated on an Axis-ultra Dld-600 W (Kratos, Kawasaki, Japan) photoelectron spectrometer. Scanning electron microscopy (SEM, JSM-IT800, JEOL, Akishima, Japan) was used to examine the morphology of MS NPs with an operating voltage of 5 kV. The presence of residual CTAC in the nanoparticle samples was tested by FT-IR. The FT-IR measurements were conducted using the potassium bromide (KBr) pellet method. Before tableting, 200–300 mg of the dried KBr and 1–1.5 mg of dried samples were thoroughly ground in an agate mortar. Then the mixture was compressed into a pellet and analyzed over a scanning range of 400 cm^−1^–4000 cm^−1^.

### 2.7. DLS Measurements

The hydrodynamic diameters of nanoparticles, both untreated and treated with 55% FBS, were analyzed using DLS measurements. All dynamic light scattering (DLS) experiments were performed on a Zetasizer Nano ZS90-PV (Malvern Instruments, Malvern, UK) at 25 °C with a wavelength of 830 nm and a scattering angle of 90°. The NPs (1 mg) were weighed, ultrasonically dispersed in distilled water (1 mg/mL), and transferred into a 1.5 mL cuvette after dispersion. Data were collected for 20 s, and results from three sets of 20 replicates were averaged. Zeta potentials of NPs were also detected on a Zetasizer Nano ZS90-PV (Malvern Panalytical, Malvern, UK). Data analysis was performed using Zetasizer Software (Version 7.13). Each DLS experiment was repeated three times.

### 2.8. GSH Depletion and Determination of Thiol Content on the Surface of MS NPs

GSH depletion: The indicator DTNB was utilized to investigate GSH consumption induced by the -S-S-S-S- in nanoparticles. In brief, 1 mL of PBS nanoparticle dispersions at concentrations of 1, 2, 4, 6, and 8 mg/mL were each mixed with 1 mL of a 0.8 mg/mL GSH solution in PBS, incubated at 37 °C for 24 h, and then centrifuge at 12,000 rpm for 15 min. In each group, 0.75 mL of the supernatant was added to 3.25 mL of distilled water. Then 600 μL of the supernatants were added to 2.4 mL of a DTNB solution (0.02 mg/mL in Tris HCl buffer) and incubated for 5 min. Finally, the absorbance in the range of 200–800 nm was measured using UV-vis spectroscopy (Lambda 365, Perkin Elmer, Shelton, CT, USA) to determine the extent of GSH consumption.

Determination of thiol content on the surface of MS NPs: In this experiment, different nanoparticles were incubated directly with DTNB. Briefly, samples containing 1, 2, 4, 6, and 8 mg of each type of nanoparticle (including non-dried SMONs, dried-SMONs, HSMONs, and CMONs) were dispensed into separate tubes. Subsequently, 2 mL of DTNB solution (0.02 mg/mL) was added to each tube containing nanoparticles, yielding final MS NP concentrations of 0.5, 1, 2, 3, and 4 mg/mL. After reacting for 10 min, the mixtures were first photographed and then centrifuged again (12,000 rpm, 30 min). The final supernatants were collected, and the absorbance of TNB^2−^ at 412 nm was measured by UV-Vis spectroscopy.

### 2.9. Biodistribution and In Vivo Image

In order not to change the method of covalent coupling of GA to MS NPs, Cy7 SE/Cy3 SE was used to replace GA in this study. Female Balb/c mice, aged 6 weeks, were injected intravenously (*i.v.*) with 100 μL of Cy7 SE (Cy3 SE) @MS NPs at a dosage of 2.5 μg Cy7/Cy3 per mouse, followed by near-infrared fluorescence (NIRF) imaging using a Pearl^®^ Trilogy small animal imaging system (LI-COR Biotech, Lincoln, NE, USA). For fluorescence imaging of tissue distribution, 24 h after intravenous injection, mice (*n* = 2–4) were euthanized, and all major organs, including the heart, liver, spleen, lung, and kidney were carefully collected. The following excitation (λ_ex_ = 710–760 nm) and emission (λ_em_ = 810–875 nm) filters were used. The same illumination settings were applied for all image acquisitions, and fluorescence and photographic images were acquired and merged. Pseudo-color images represent the spatial distribution of fluorescence intensity in organs. Concurrently, fresh lung tissues were rapidly harvested to avoid postmortem tissue changes. The tissue blocks were laid flat, and then quickly frozen for sectioning. The tissue sections were incubated with an immunofluorescence staining blocking solution for 1 h. Subsequently, a 1:200 dilution of rabbit CD31 primary antibody was dropped onto the tumor tissue sections and incubated at 4 °C overnight. Afterward, the sections were washed with an immunofluorescence staining detergent for 5 min per wash, and this was repeated 4 times. Then, a 1:100 dilution of FITC-conjugated goat anti-rabbit secondary antibody was dropped and incubated at room temperature for 1 h. The sections were stained with a DAPI working solution (10 μg/mL) and stained at room temperature for 10–20 min. Once the DAPI was complete, 5–10 μL of an anti-fluorescence quenching mounting medium or neutral gum was dropped, and the sections were covered with a clean cover slip and sealed. The CD31-labeled blood vessels in the lung tissue were then observed under a confocal microscope (λ_ex_ = 490 nm, λ_em_ = 525 nm). To observe the position of MS NPs-Cy3 SE in lung tissue, the excitation wavelength was adjusted to λ_ex_ = 652 nm, and the emission wavelength to λ_em_ = 668 nm, using either the confocal microscope or a slice scanner. The prepared slides were stored in a slide box at 4 °C in a refrigerator, where they can be preserved for about one week.

### 2.10. Preparation of Protein Corona-Coated MS NPs

First, 500 μg of each sample was suspended in 1 mL of PBS solution containing 55% FBS and incubated at 37 °C for 1 h to ensure thorough mixing, which facilitates the formation of protein corona on the surface of MS NPs. This concentration is determined based on in vivo biological conditions. Following incubation, the unbound proteins were removed by centrifugation at 13,000 rpm for 20 min, a process repeated three times with PBS. To isolate the protein corona, the purified protein corona-coated MS NPs were first dispersed in 50 μL of 10% SDS solution (10 mg/mL). They were then subjected to ultrasonication for 30 min in an ultrasonic bath, flowed by centrifugation. The supernatant was then collected. The purified protein corona-coated MS NPs were stored at 4 °C for future use.

### 2.11. Isolation and Purification of Surface Adsorbed Proteins

To isolate the protein corona, the purified protein corona-coated MS NPs were first dispersed in 50 μL of 10% SDS solution (10 mg/mL). They were then subjected to ultrasonication for 30 min in an ultrasonic bath, flowed by centrifugation. The resulting supernatant, which contained the protein corona solution, was obtained for further protein quantification. To purify the proteins from the SDS for LC-MS/MS identification, 950 μL of trichloroacetic acid (TCA) solution (10% *w*/*v* in acetone) was firstly added to the supernatant. Proteins were precipitated from the solution by storing at −80 °C overnight and then centrifuged at 13,000 rpm for 15 min to form a pellet. The supernatant was discarded, and 500 μL of sodium deoxycholate (Fluka) at 0.03% *w*/*v* in water and 100 μL of trichloroacetic acid (72% *w*/*v* in water) was added. The mixture was vortexed thoroughly and incubated on ice for 1 h to allow for protein re-precipitate proteins, followed by centrifugation at 13,000 rpm for 15 min. The supernatant was removed, and 950 μL of acetone was added to the pellet to precipitate the proteins again at −80 °C for 1 h. The samples were centrifuged at 13,000 rpm for 15 min to pellet precipitated proteins. The supernatant was discarded, and the remaining acetone was air-dried for approximately 30 min.

### 2.12. Sodium Dodecyl Sulfate−Polyacrylamide Gel Electrophoresis (SDS-PAGE) and Coomassie Brilliant Blue Staining

The total amount of adsorbed protein was determined by the BCA method. In total, 20 μL of enriched liquid and 200 μL of BCA reaction solution were added to a 96-well plate and incubated for 30 min at 37 °C. The remaining 30 μL of the enrichment solution was utilized to identify the adsorbed proteins via SDS-PAGE gel electrophoresis. An aliquot (30 μL) of the protein corona solution was mixed with an equal volume of Laemmli buffer (2x), denatured at 90 °C for 10 min, and then cooled to 4 °C to halt the reaction. After the droplets on the wall were collected by centrifugation at 5000 g for 3 min, 20 μL of the mixture was subjected to 12% SDS-PAGE gel electrophoresis. The electrophoresis was run at a constant voltage of 100 V with a current of 50 mA until the blue indicator approached the bottom of the gel. After electrophoresis, a coomassie brilliant blue staining kit was employed to visualize the protein bands on the gel.

### 2.13. Preparation of Protein Corona for LC-MS/MS Analysis

For LC-MS/MS analysis, the purified proteins underwent further enzymatic digestion and alkylation. First, 45 μL of 100 mM ammonium bicarbonate (NH_4_HCO_3_) was added to each sample. Then, 5 μL of acetonitrile and 100 mM DTT in 100 mM NH_4_HCO_3_ were added to the samples. The proteins were incubated at 37 °C for 1 h to ensure complete reduction. The samples were then cooled to room temperature, and 5 μL of the alkylating agent (500 mM of iodoacetamide in 100 mM NH_4_HCO_3_) was added. The samples were then incubated in the dark for one hour to allow the cysteine group to be alkylated. For protein digestion, 2 μL of a 0.25 µg/µL trypsin solution was added to each sample, and the digestion was carried out overnight at room temperature. Finally, 5 μL of 20% *v*/*v* formic acid was added to the sample to adjust the pH to 1. The resulting samples were subsequently analyzed using electrospray liquid chromatography–tandem mass spectrometry on an Orbitrap Fusion Lumos mass spectrometer (LC-MS/MS), which was coupled online to an EASY-nLC 1200 system (Thermo Fisher Scientific, San Francisco, CA, USA).

### 2.14. In Vivo Anti-Metastatic Efficacy

Female Balb/c mice, aged 5 weeks, were intravenously (*i.v.*) injected with 1 × 10^5^ 4T1-luc cells suspended in 100 μL of PBS to generate the lung metastasis model for breast cancer. After tail injection of 4T1-luc cells (0 d), the mice were randomly divided into four groups: Control, Free GA, GA@CMONs and GA@HSMONs, and *i.v.* injected, respectively, with 200 μL of PBS, Free GA, GA@CMONs, and GA@HSMONs (containing 4 mg/kg of GA) at days 7, 10, and 13 post injection. The mice were intraperitoneally (i.p.) injected with D-luciferin (150 mg/kg) and the bioluminescent imaging (BLI) signals were captured 10 min later using the IVIS Lumina LT Series III (Perkin Elmer IVIS Spectrum, Waltham, MA, USA) to monitor the growth of lung metastatic tumors. At day 15, the mice were sacrificed, and the lung tissues of each group were removed and photographed using a digital camera. The BLI intensity of both the mice and the lung tissues was quantified using the IVIS Lumina III software (Version 4.4).

### 2.15. In Vivo Biological Safety

To assess the in vivo biosafety, twenty Balb/c mice (female, 6–8 weeks) were randomly divided into four groups: PBS, free GA, GA@CMONs, and GA@HSMONs. From day 0, each group received tail vein injection every 3 days for a total of 3 doses. On day 10, blood was collected from the retro-orbital plexus before the mice were sacrificed, and major organs, including the heart, liver, spleen, lung, and kidneys, were collected and sectioned. For hematological analysis, blood samples were allowed to clot for 2 h before being centrifuged at 3000× *g* for 20 min to separate the serum. Key blood indicators, including alanine aminotransferase (ALT) and aspartate aminotransferase (AST), along with renal function markers creatinine (CRE) and blood urea nitrogen (BUN), were quantified using a microplate reader (BioTek, Winooski, VT, USA). Histological examination involved staining formalin-fixed tissues with hematoxylin and eosin (H&E) to assess pathological changes in the major organs. Body weight measurements of the mice were recorded throughout the in vivo anti-metastatic study. All animal experiments were conducted with approval from the Institutional Animal Care and Use Committee of Huazhong University of Science and Technology (IACUC number: 3348).

### 2.16. Statistical Analysis

All statistical analyses were performed using the GraphPad Prism 10.3.0 software package (GraphPad Prism, Inc., San Diego, CA, USA). All error bars used in this study are mean ± standard deviation (SD) of at the least three independent experiments. Statistically significant *p* values are indicated in figures and/or legends as *** *p* < 0.001; ** *p* < 0.01; * *p* < 0.05, and n.s. represented no significance.

## 3. Results and Discussion

### 3.1. Synthesis and Characterization of MS NPs

**Figure 1:**
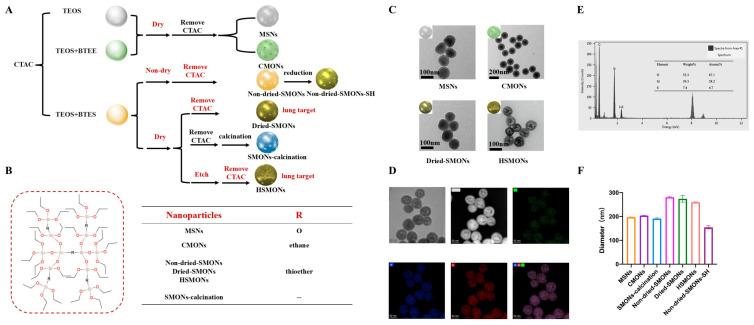
Synthesis and characterization of MS NPs. (**A**) Illustration for the preparation process of different MS NPs. These arrows and the descriptions above them represent the synthesis process of different MS NPs. (**B**) The chemical homology principle for constructing the hybrid silsesquioxane framework of MS NPs. R stands for O/ether/thioether groups. (**C**) TEM images of MS NPs. (**D**) FTEM images of HSMONs. Scale bar: 50 nm. (**E**) Energy-dispersive X-ray spectroscopy (EDX) of the O, Si, and S of HSMONs. (**F**) Hydrodynamic sizes of MS NPs.

Mesoporous silica nanoparticles were synthesized using TEOS, BTEE, and/or BTES as silica source precursors via a CTAC-directed sol–gel process. A series of mesoporous silica-based nanoparticles (MS NPs) were prepared, including inorganic mesoporous silica nanoparticles (MSNs), -C-C-bridged mesoporous organosilica nanoparticles (CMONs), dried thioether-bridged mesoporous organosilica nanoparticles (dried-SMONs), dried-SMONs without desiccation (non-dried-SMONs), non-dried-SMONs treated with triphenylphosphine to obtain thiol groups (non-dried-SMONs-SH), calcined SMONs (SMONs-calcination), and thioether-bridged deformable hollow mesoporous organosilica nanoparticles (HSMONs). Schematic illustrations of these nanoparticles are presented in Figure 1A,B.

TEM, SEM, and FTEM were employed to examine the morphology of the nanoparticles. TEM and SEM images in Figure 1C and Figure S1A–E showed that all of these MS NPs were uniformly dispersed with spherical shapes. Only the HSMONs exhibited unique hollow spheres due to the etching step. The average diameters of MSN, CMONs, dried SMONs, non-dried-SMONs, and HSMONs were approximately 100 nm (Figure 1C and Figure S1A,C–E). After removing the organic components from the SMON structure, the diameter of SMON calcination reduced to nearly 80 nm (Figure S1B). X-ray photoelectron spectroscopy (XPS) analysis has been conducted to confirm the chemical composition of MS NPs (Figure S1F–H). To further ascertain the presence of sulfur in the HSMONs’ framework, FTEM was conducted, showing uniform distribution of all elements throughout the nanoparticles (Figure 1D). The detection of sulfur signals confirmed the successful integration of tetrasulfide bonds in HSMONs, with elemental mapping indicating a sulfur content of about 6.8% ± 0.55% in HSMONs (Figure 1E).

Dynamic light scattering (DLS) measurements confirmed that MS NPs in deionized water were well-dispersed and negatively charged (before modification, as shown in Figure S2A), with hydrodynamic sizes of MSNs, CMONs, non-dried-SMONs, SMONs-calcination, non-dried-SMONs-SH, dried-SMONs, and HSMONs determined to be 196.9 ± 2.5 nm, 203.4 ± 3.9 nm, 190.8 ± 5.6 nm, 280.5 ± 4.7 nm, 154.4 ± 11.1 nm, 273.9 ± 23.6 nm, and 259.8 ± 3.7 nm, respectively (Figure 1F). When dispersed in serum, the hydrodynamic sizes of the nanoparticles remained within the nanoscale range (Figure S2B), indicating that these diverse MS NPs could maintain their nanoscale dimensions in biological environments. This phenomenon indicated that these different MS NPs could remain nano-scaled when intravenously injected into the tail vein of the mouse.

The pore structures of the nanoparticles were further characterized through nitrogen sorption analysis. As demonstrated in Figure S3, all samples displayed a typical IV-type isotherm, indicative of an abundance of mesopores. It can be seen from the Barrett–Joyner–Halenda (BJH) pore size distribution curves that HSMONs maintain a pore size of 4.2 nm, which is larger than 2.7 nm for MSNs and 2.5 nm for CMONs (Table S1). FT-IR spectra were utilized to confirm the effective removal of surfactant (CTAC) from the nanoparticles, with spectra showing a clear difference before (the purple line) and after a series of repetitive washing steps (the red line, gray line, and yellow line).

A free CTAC sample (green line) was used as a control in our analysis. The major FT-IR peaks indicative of CTAC, namely the -CH_2_ stretching vibrations at 2925 cm^−1^ and 2854 cm^−1^, and the -C-H scissoring vibration at 1450 cm^−1^, were marked with dotted lines in Figure S4. The absence of these characteristic -CH- peaks of CTAC in the nanoparticles after the washing process indicated the efficient removal of the surfactant.

Following synthesis and characterization, the MS NPs were conjugated with gambogic acid (GA) as depicted in Figure S5A,B. The coupling ratio and drug loading efficiency of GA were determined by measuring the absorbance at the wavelength corresponding to the maximum absorption of GA using a microplate reader. The results showed that the coupling rates for GA@HSMONs and GA@CMONs were 36.98% ± 3.94% and 29.86% ± 1.25%, respectively, while their drug loading rates were10.06% ± 0.08% and 7.54% ± 0.02%, respectively (Table S2). Both types of MS NPs exhibited a good coupling efficiency, which was beneficial to subsequent in vivo experiments.

### 3.2. Tetresulfide-Contented MS NPs Induced GSH Consumption

It is generally acknowledged that tumor cells contain significant higher levels of glutathione (GSH) compared to normal cells [8,27]. The presence of tetrasulfide bond can endow the nanoparticles with GSH-response degradation properties [35]. To further investigate GSH depletion by MS NPs, we employed DTNB to quantify GSH content. Under mild alkaline conditions (pH 7–8), DTNB reacts with the –SH groups on GSH, generating the 2-nitro-5-thiobenzoate anion (TNB^2−^), which exhibits a strong yellow color at 412 nm. In our cases, GSH can be oxidized due to the redox-active properties of the -S-S-S-S- moieties within the framework of MS NPs.

When GSH concentration was fixed at 0.8 mg/mL, GSH depletion was monitored after its reaction with different concentrations of MS NPs. As shown in Figure S6, an increase in the concentration of non-dried-SMONs, dried-SMONs, and HSMONs corresponded to a decrease in GSH levels. On the contrary, the GSH consumption in the solution after incubation with CMONs was almost the same as that in the bare GSH solution, demonstrating that the -S-S-S-S- group containing nanoparticles led to GSH consumption. The greater the degree of GSH oxidization, the less reactive GSH remains, resulting in lower absorbance at 412 nm. Therefore, a lower absorbance at 412 nm indicates a higher level of GSH consumption. Notably, the extent of GSH depletion by nanoparticles, as observed in Figure S6, followed the order non-dried-SMONs > dried-SMONs > HSMONs > CMONs. Therefore, it could be concluded that non-dried SMONs, dried SMONs, and HSMONs were capable of depleting GSH.

### 3.3. Organ Tropism of MS NPs

**Figure 2:**
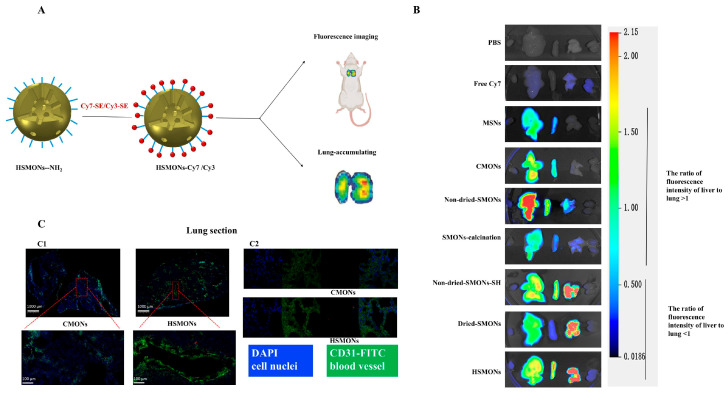
In vivo biodistribution of MS NPs. (**A**) Cy7 SE/ Cy3 SE was coupled to MS NPs as fluorescent dyes. (**B**) Fluorescence images of free Cy7 SE and Cy7SE@MS NPs in major organs 24 h after tail vein injection. (**C**) Immunofluorescence staining of lung frozen sections 24 h after tail vein injection, and immunofluorescence staining of the same sections to examine the presence of blood vessels (green for CD31-FITC staining) and the presence of nuclei (blue for DAPI staining). (**C1**) Slice scanner. Scale Bar: upper panel: 1000 μm; lower panel 100 μm. (**C2**) Confocal microscope (magnification 40×).

Following synthesis and characterization, MS NPs were conjugated with a fluorescent dye Cyanine 7 SE (Cy7 SE) and intravenously injected into mice for biodistribution monitoring (Figure 2A). At 24 h post injection, the integrated density (=mean fluorescence intensity × area) of the heart, lung, liver, kidney, and spleen was analyzed quantitatively. As shown in Figure 2B, at the 24 h mark, mice treated with free Cy7 SE exhibited no detectable fluorescence, demonstrating that free dye was rapidly metabolized out of the body. For the groups treated with MSNs and CMONs, the liver exhibited significantly higher fluorescence intensity compared to the lungs. Conversely, in the HSMON treatment group, the liver’s fluorescence intensity was markedly lower than that of the lungs.

Initially, we speculated that the tetrasulfide bonds played an important role in the preferential accumulation of NPs in the lungs. To test this, dried-SMONs (which were the HSMONs before the etching step) were used to monitor biodistribution. As shown in Figure 2B, the lung exhibited a notably higher fluorescent intensity compared to the liver. However, after the calcination process (SMONs-calcination), which eliminated all organic components, the liver preference in biodistribution reappeared, suggesting that -S-S-S-S- bonds might influence the in vivo fate of the nanoparticles. Then, we made an unexpected observation: when SMONs were not dried under vacuum conditions (referred to as non-dried-SMONs), the lung-preferred biodistribution shifted back from a lung preference to a liver preference. This finding indicated that -S-S-S-S- bonds were not the key point for lung tropism, as both dried-SMONs and non-dried-SMONs containing the -S-S-S-S- bond displayed different biodistribution patterns.

We then speculated that -SH content may change during the drying processes. To validate this hypothesis, the non-dried-SMONs, which exhibited a preference for liver tissue, were treated with triphenylphosphine to convert the tetrasulfide bonds into -SH groups. The resulting nanoparticles were designated as non-dried-SMONs-SH. These reduced nanoparticles were then labeled with Cy7 SE and injected via the tail vein of BALB/c mice. As depicted in Figure 2B, the accumulation of thiolated nanoparticles in the lungs was indeed higher than that in the liver. Notably, the liver-to-lung integrated density ratios for the MSN, CMON, non-dried SMON, and SMON calcination groups were 12, 16, 3.1, and 3.8, respectively (all > 1), indicating predominant liver tropism. In contrast, the non-dried SMON-SH, dried SMON, and HSMON groups exhibited ratios of 0.78, 0.6, and 0.74, respectively (all < 1), suggesting a shift toward lung accumulation.

Confocal fluorescence microscopy was also performed to visualize the detailed distribution of MS NPs. As shown in Figure 2C, the frozen sections of mouse lung tissue injected with Cy3 SE-labeled MS NPs after immunofluorescence staining further confirmed that HSMONs were preferentially delivered to lung tissue compared to CMONs (Figure 2C).

### 3.4. Organ Selectivity Is Correlated with Thiol Content on the Surface of MS NPs

**Figure 3:**
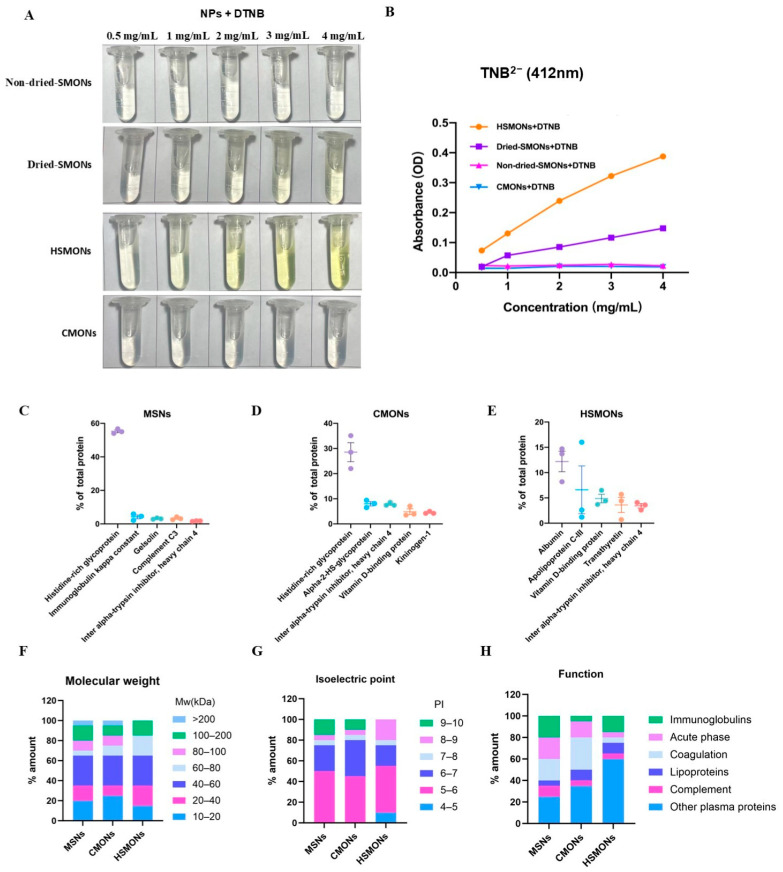
Detection of -SH in nanoparticles and proteomics study of protein coronas formed on MS NPs. (**A**) Detection of -SH in MS NPs using DTNB. (**B**) UV-vis absorption of different samples in (**A**). Quantification of the percentage of total proteins of the top five protein components in the protein corona of MSNs (**C**), CMONs (**D**), and HSMONs (**E**). The top 20 most abundant corona proteins were categorized based on their calculated molecular weight (**F**), isoelectric point (**G**), and biological function (**H**).

The -SH content in different nanoparticles was also determined to confirm that the thiol group, rather than the -S-S-S-S- bonds, played a vital role in lung tropism. DTNB, known for detecting the -SH groups, was also utilized to assess the -SH content on MS NPs. Different concentrations of CMONs, non-dried-SMONs, dried-SMONs, and HSMONs were directly dispersed in a 0.02 mg/mL DTNB solution. Since CMONs lacked sulfur, they could not react with DTNB. After the reaction of the latter three types of MS NPs, it was observed that there was no color change in the non-dried-SMON-treated tube (Figure 3A), indicating that non-dried-SMONs only contained tetrasulfide bonds, and so would not react with DTNB. However, when incubated with DTNB, both the dried-SMON- and HSMON-treated groups turned yellow, indicating that some tetrasulfide bonds had been reduced to -SH, and the content of -SH in the HSMONs was greater than that of dried-SMONs (Figure 3A,B). This assay revealed that the -SH group was indeed accidentally introduced to dried-SMONs compared with non-dried-SMONs, likely due to partial reduction in tetrasulfide bonds during the drying process.

The lung tropism observed in HSMONs, dried-SMONs, and non-dried-SMON-SH may be attributed to the presence of the -SH group on the surface of mesoporous silica nanoparticles. The hydrophobicity and surface SH of MS NPs may affect their binding affinity to plasma proteins. Further analysis was required to identify the definitive types of protein corona formed.

### 3.5. The Organ-Selectivity Is Correlated with Proteins Corona Absorbed on MS NPs

It is well known that nanoparticles rapidly adsorb proteins and form protein corona on their surface following intravenous administration. The formation of this protein corona can greatly change the surface properties and biological interactions of nanoparticles, thereby influencing their in vivo fate. Therefore, differences in the proteins adsorbed on the surface of MS NPs after entering the systemic circulation might result in different consequences. To verify our hypothesis, MSNs, CMONs, and HSMONs were incubated with BALB/c mouse serum. Following washed with PBS (Figure S7A–C), the protein coronas adsorbed on the surface of the nanoparticles were isolated and analyzed using both SDS-PAGE and LC-MS/MS.

As shown in the SDS-PAGE image (Figure S7D), the liver-selective MSNs and CMONs exhibited similar protein band patterns. Compared to the lung-selective HSMONs, they had a greater variety of protein corona components and less protein at the 70 kDa band. In contrast, for HSMONs, the 70 kDa band was much darker than in the other two liver-selective MS NPs groups.

To further identify the precise types of proteins absorbed on the surface of nanoparticles, proteomics was performed to analyze the composition of proteins. Using LC-MS/MS, 666, 626, and 539, proteins absorbed on the MSNs, CMONs, and HSMONs, respectively, were identified and quantified (Figure S8A). Subsequently, the top 20 proteins for each type of MS NP were listed in Table 1, and the top 5 proteins, which are likely to play a predominant role in the formation of the protein corona, were quantified in Figure 3C–E.

Histidine-rich glycoprotein (HRG) was identified as the most abundant protein in both liver-preferred MSNs and CMONs, constituting approximately 30% and 60% of the total protein content, respectively. It has been reported that HRG binds to N-sulfated polysaccharide chains on the surface of liver endothelial cells [36,37,38], which might be the reason why HRG-absorbed MS NPs preferred liver accumulation. On the contrary, for the lung-preferred HSMONs, the top protein was albumin, constituting about 13% of the total proteins. Notably, the albumin content in the MSNs and CMONs groups was dramatically lower compared to the HSMON group (Figure S8B). The -SH group present on the surface of HSMONs might interact with the -SH group (Cys-34) on the surface of albumin when nanoparticles are incubated with mouse serum. Free -SH groups on the nanoparticle surface may undergo a disulfide exchange reaction with the -SH group of albumin’s Cys-34, forming a covalent -S-S- bond that firmly anchors the albumin to the nanoparticle. Even without forming a stable disulfide bond, weak interactions via a thiol-disulfide complex might occur between them. Furthermore, albumin is well-known to undergo conformational changes when interacting with hydrophobic surfaces, enhancing its adsorption stability [39,40]. Given that other plasma protein rarely contains the free -SH group, it is plausible that -SH-rich NPs could effectively recruit plenty of endogenous albumin from the mouse serum. Consequently, it was reasonable for HSMONs to absorb a considerable amount of albumin compared to the other two MS NPs, which lack -SH groups. M. Schaffler demonstrated that conjugation with blood proteins, such as albumin and ApoE remarkably reduced the retention of gold nanoparticles in the liver. Albumin-coated gold NPs displayed greater accumulation in the lungs and brain than ApoE-GNP [41]. We speculated that albumin played a critical role in the lung tropism, at least for mesoporous silica nanoparticles. Among the top 5 proteins in the three groups, only inter-alpha-trypsin inhibitor, heavy chain 4, was shared. Further investigation is necessary to determine whether the organ tropism of nanoparticles depends on only one typical protein or a combination of several key proteins.

Consequently, the top 20 absorbed proteins were categorized based on their molecular weight [28]. As depicted in Figure 3F, 5% of the proteins in both the MSNs and CMONs groups were larger than 200 kDa, and 10% fell within 80–100 kDa range, while none of the proteins in these two molecular weights ranges existed in the HSMONs group. In contrast, the HSMONs group had a higher proportion of proteins in the 60–80 kDa range compared with the other two liver-selective groups.

In addition, the top 20 proteins were classified according to their isoelectric points (pI). As depicted in Figure 3G, liver-selective MSNs and CMONs exhibited similar pI compositions, with a range of 5–6, 6–7, 7–8, 8–9, and 9–10. In contrast, lung-selective HSMONs lacked proteins at pI 9–10 and instead featured proteins at pI 4–5. And proteins at pI 8–9 absorbed on HSMONs are more than those of MSNs and CMONs. We also observed that nearly 80% of the absorbed proteins were negatively charged under the physiological condition (pH = 7.4) for all three groups. Since these particles were modified using the same strategy (APTES modification), the zeta potential of these particles was positively charged. So, the typical pI profile of HSMONs was possibly due to their unique composition of -S-S-S-S- and -SH. And for MSNs and CMONs, the -C- C-composition added in CMONs did not change the serum affinity. The biological functions of the proteins were also analyzed, as shown in Figure 3H, and nearly 20% of the total proteins belonged to acute phase and coagulation for MSNs and CMONs, a proportion significantly higher than that observed for HSMONs. Meanwhile, HSMONs contained the highest percentage of other plasma proteins when compared to MSNs and CMONs.

In summary, the variation in types, content, molecular weight, surface charge, and biological function of proteins on the surface of the MS NPs may greatly affect their surface properties, physiological functions, and biodistribution.

### 3.6. Lung-Selective HSMONs Induced Enhanced Anti-Tumor Activity

Liver selective CMONs and lung-selective deformable HSMONs were chosen for the following experiment. GA was conjugated to these nanoparticles using the same approach as for Cy7 SE/Cy3 SE. The reason for choosing deformable HSMONs was that they contained the highest sulfur content among the lung-selective nanoparticles, and the hollow structure further increased the drug loading efficiency. In addition, the deformable structure of HSMONs enhanced the cellular uptake via a spherical-to-oval morphology change.

**Figure 4:**
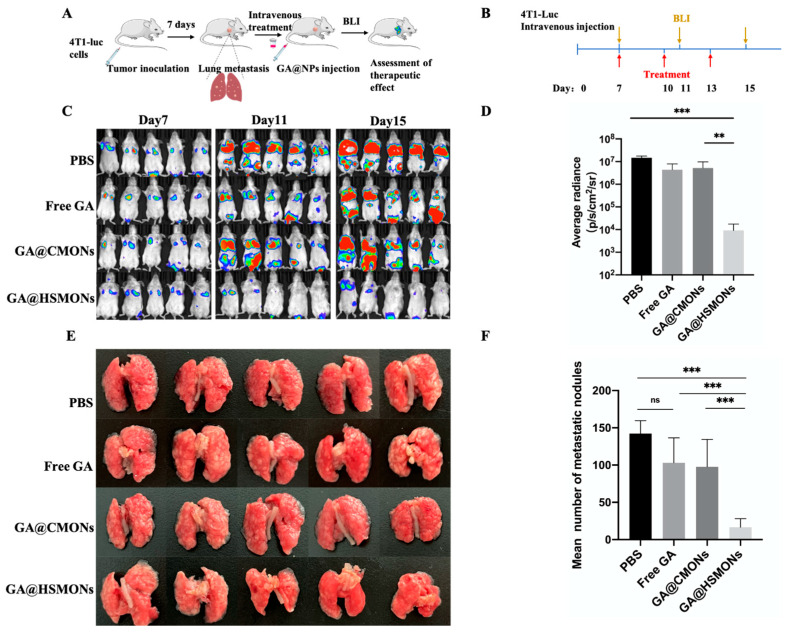
Effect of inhibiting lung metastasis in vivo. (**A**,**B**) Schematic diagram of the treatment schedule. The red arrows represent the injection times for the nanoparticles, and the brown arrows correspond to the times when bioluminescent imaging was performed in mice. (**C**) Bioluminescence images of lung metastasis at different time points. (**D**) According to the bioluminescence signal intensity on day 15, the degree of lung metastasis in different treatment groups was compared. (**E**) Pictures of pulmonary nodules of 15-day lung tissue in different groups (4% paraformaldehyde fixation). (**F**) The average number of metastatic nodules in lung tissue of each group on day 15. (*n* = 5, n.s. represented no significance, ** *p* < 0.01, *** *p* < 0.001.)

The in vivo therapeutic efficacy was evaluated in a lung metastasis model of breast cancer. Figure 4A,B illustrate the procedure for the animal experiments. The treatment began on day 7 following the inoculation of 4T1 cells with luciferase gene expression (4T1-luc cells, Figure S9) via the tail vein. During the therapeutic period, the luciferase signals from 4T1-luc cells were monitored using noninvasive bioluminescence imaging to assess lung metastasis. The bioluminescence images of Balb/c mice from each group and the quantitative analysis of bioluminescence intensity at day 15 is shown in Figure 4C,D (5 representative mice), Figure S10A,B (*n* = 7–9). Notably, the GA@HSMONs treatment group demonstrated a significant inhibitory effect (*p* < 0.001) on lung metastasis, with nearly no signal detected compared with the PBS, Free GA, or GA@CMONs groups. At the endpoint, the mice were euthanized, and lung tissues from each group were carefully excised and photographed using a digital camera. The mean number of macroscopic metastatic nodules was quantified (Figure 4F). In comparison to the other groups, treatment with GA@HSMONs dramatically decreased the incidence of lung metastasis, exhibiting the fewest metastatic nodules (Figure 4E and Figure S10C,D, *p* < 0.001). This result underscores the potent antitumor activity of GA@HSMONs, highlighting their efficacy in curbing metastatic spread.

It is speculated that several factors contribute to the remarkable potency of deformable HSMONs in inhibiting lung metastasis. (1.) HSMONs were capable of targeting the lung tissue; (2.) since LC-MS/MS identified albumin as the most abundant protein on the surface of HSMONs, after coating with albumin, the albumin-coated HSMONs tended to target tumors via transcytosis, traversing the vasculature endothelial cells through the gp60 receptor expressed on the endothelial cell membranes, a mechanism that may be analogous to that of Abraxane [24,25,26,27]; (3.) the deformable nature of HSMONs was able to enhance the cellular uptake compared to their rigid MS NP counterparts [5,8,42]; and (4.) upon endocytosis by tumor cells, the elevated GSH levels within these cells can induce the degradation of HSMONs, thereby releasing GA intracellularly [8,28,43,44]. Conversely, CMONs lacked the aforementioned tumor-targeting advantages, which led to the rapid growth of tumors in the lung. Collectively, the natural lung tropism of protein corona-absorbed HSMONs, albumin-mediated transcytosis to the tumor cells, and GSH-triggered GA release account for their potent ability to inhibit tumor growth in the lung.

### 3.7. In Vivo Toxicity

In the in vivo anti-lung metastasis experiment, there was no significant difference in the weight change trend of mice among these groups (Figure S11). The systemic toxicity of the fabricated MS NPs was evaluated in a normal mouse following intravenous injection, using blood tests and histological staining assays. The hepatic and renal functions were evaluated through the measurement of the ALT, AST, CRE, and BUN levels, respectively. In this study, the three treatment groups did not exhibit significant differences in the ALT, AST, BUN, and CRE, compared with the PBS control group (Figure S12). This implied appropriate maintenance of normal hepatic and renal functions after the intravenous injection of the MS NPs. Additional toxic potentials of the prepared MS NPs were evaluated by histological staining of the heart, kidney, liver, lung, and spleen tissues (Figure S13). It is worth noting that no considerable difference in the morphology of each organ was observed between the control and the other three treatment groups. The results of both the blood assay and histological staining test indicated the safe application of GA@HSMONs for the treatment of lung metastasis in breast cancer.

## 4. Conclusions

In summary, a series of organ-selective MS NPs was successfully synthesized and systemically administered to mice. Our findings elucidated that the structure and the pore size were not the critical points, in our cases, for determining the final in vivo destination. Contradicting our expectations, the existence of the -SH group was proved to change the organ tropism, at least for MS NPs. The proteomic data showed that liver-preferred MS NPs absorbed the highest amount of HRG in the serum, while the lung-preferred MS NPs, such as HSMONs, recruited the highest amount of albumin. Albumin-absorbing and the intrinsic -S-S-S-S- bond in the framework of deformable HSMONs made it long-circulating in the blood and led to lung tropism; after arriving at lung tissue, HSMONs were largely endocytosed to tumor cells due to their deformability. The high GSH level inside the tumor cells triggered the degradation of HSMONs, leading to the release of GA mainly inside the tumor cells. The specific protein corona on the surface of nanoparticles was only part of the reason that affected specific tissue targeting. In future studies, we will continue to explore the structure and the subsequent biological influences.

## Figures and Tables

**Table 1:** Top 20 proteins absorbed on the surface of different MS NPs.

MSNs (%)	CMONs (%)	HSMONs (%)
Histidine-rich glycoprotein (55.24)	Histidine-rich glycoprotein (28.39)	Albumin (12.20)
Immunoglobulin kappa constant (4.21)	Alpha-2-HS-glycoprotein (8.00)	Apolipoprotein C-III (6.63)
Complement C3 (3.29)	Inter alpha-trypsin inhibitor, heavy chain 4 (7.80)	Vitamin D-binding protein (4.87)
Gelsolin (3.18)	Vitamin D-binding protein (4.83)	Transthyretin (3.58)
Albumin (1.78)	Kininogen-1 (4.42)	Inter alpha-trypsin inhibitor, heavy chain 4 (3.43)
Inter alpha-trypsin inhibitor, heavy chain 4 (1.73)	Complement C3 (4.02)	Complement C3 (2.58)
Platelet factor 4 (1.63)	Actin, cytoplasmic 1 (3.53)	Immunoglobulin heavy constant gamma 2B (2.46)
Plasminogen (1.55)	Immunoglobulin kappa constant (2.96)	Mitogen-activated protein kinase kinase kinase 2 (1.96)
Kininogen-1 (1.14)	Albumin (2.24)	Apolipoprotein A-IV (1.91)
Immunoglobulin heavy constant gamma 2C (Fragment) (0.87)	Serum amyloid P-component (1.44)	Alpha-2-HS-glycoprotein (1.86)
Apolipoprotein E (0.85)	Serum amyloid A-1 protein (1.42)	Proteoglycan 4 (1.78)
Chemokine subfamily B Cys-X-Cys (0.75)	Apolipoprotein A-II (1.39)	Kininogen-1 (1.57)
Complement factor H (0.63)	Apolipoprotein A-IV (1.29)	Actin, cytoplasmic 1 (1.57)
Vitamin D-binding protein (0.59)	Gelsolin (0.99)	Immunoglobulin heavy constant gamma 2C (Fragment) (1.52)
Antileukoproteinase (0.52)	Fibronectin (0.99)	Serum amyloid P-component (1.48)
Immunoglobulin heavy constant mu (0.49)	Chemokine subfamily B Cys-X-Cys (0.85)	Hemopexin (1.45)
Actin, cytoplasmic 1 (0.48)	Platelet factor 4 (0.85)	Immunoglobulin kappa constant (1.30)
Fibronectin (0.45)	Plasma kallikrein (0.55)	Keratin, type II cytoskeletal 2 oral (0.97)
Ig gamma-2A chain C region, A allele;Ig gamma-2A chain C region, membrane-bound form (0.44)	Plasminogen (0.52)	Carboxylesterase 1C (0.87)
Serum amyloid P-component (0.40)	Antithrombin-III (0.50)	Alpha-1-acid glycoprotein 2 (0.74)

## Data Availability

The data that support the findings of this study are available from the corresponding author upon reasonable request.

## Supplementary Materials

The following supporting information can be downloaded at: https://www.mdpi.com/article/10.3390/pharmaceutics17111389/s1, Figure S1: TEM images of MS NPs. (A,B) SEM images of MSNs (C), CMONs (D), and HSMONs (E), and XPS analysis of MSNs (F), CMONs (G), and HSMONs (H); Figure S2: (A) Zeta potential of MS NPs and (B) particle size distribution of MS NPs in FBS (*n* = 3); Figure S3: Nitrogen adsorption–desorption isotherms and pore size distribution curve of (A) MSNs, (B) CMONs, and (C) HSMONs; Figure S4: FTIR spectra of free CTAC, dried-SMNOs with surfactant removed, dried-SMNOs without surfactant removal, non-dried-SMNOs with surfactant removed, and SMON calcination; Figure S5. (A) Schematic diagram of GA carboxyl activation; (B) Surface modification and drug loading of MS NPs. In the schematic: red arrows denote the reaction procedure; blue lines stand for APTES; red dots stand for GA. Figure S6. UV-Vis spectrum of DTNB for the detection of GSH contents. GSH content after reaction with different concentrations of MS NPs (A,B,C,D) and different NPs at the same concentration (E,F,G,H,I). The initial amount of GSH was 1 mL (0.8 mg/mL), and the amount of MS NPs was 1 mL (0.5, 1, 2, 3, and 4 mg/mL); Figure S7. SDS-PAGE Analysis: (A) soft proteins of first wash from MS NPs with PBS in different groups; (B) second wash with PBS; (C) third wash with PBS; and (D) hard proteins after desorbed by 10% SDS from MS NPs. Figure S8: (A) The Venn diagrams for all identified proteins; (B) albumin/total proteins in three groups were measured by LC-MS/MS. n.s. represented no significance; *** *p* < 0.001. Figure S9: The microplate reader bioluminescence channel was used to assess the growth of 4T1-Luc cells and the bioluminescence signal of 4T1-Luc cells after the addition of D-luciferin potassium salt compared to free 4T1-luc cells and D-luciferin (*n* = 3). n.s. represented no significance; *** *p* < 0.001. Figure S10. (A) Representative bioluminescence images of lung metastasis on Day 7. (B) Representative bioluminescence images of lung metastasis on Day 15. (C) Pictures of pulmonary nodules of 15-day lung tissue in different groups (4% paraformaldehyde fixation). (D) Pictures of pulmonary nodules of 15-day lung tissue in different groups (Bouin’s fixative). *** *p* < 0.001; ** *p* < 0.01; * *p* < 0.05, and n.s. represented no significance. Figure S11: Changes in body weight of mice after different treatments (*n* = 5); Figure S12. Effect of different treatments on serum (A) BUN, (B) CRE, (C) ALT/GPT and (D) AST/GOT levels. Figure S13: H&E-stained images of major organs of mice after various treatments, including heart, liver, spleen, lung, and kidney (*n* = 3). Scale Bar = 20 μm; Table S1: Structural properties of MSNs, CMONs, and HSMONs; Table S2: Characterization of GA@NPs.

## Abbreviations

The following abbreviations are used in this manuscript:

MS NPs	Mesoporous silica nanoparticles
MSNs	Inorganic mesoporous silica nanoparticles
CMONs	-C-C-bridged mesoporous organosilica nanoparticles
dried-SMONs	Dried thioether-bridged mesoporous organosilica nanoparticles
non-dried-SMONs	SMONs without desiccation
HSMONs	Thioether-bridged hollow periodic mesoporous organosilica nanoparticles
