# Supplementary material for: The Influence of the External Chemistry of Silica-Based Mesoporous Nanocarriers on Organ Tropism and the Inhibition of Pulmonary Metastases
Source: Pharmaceutics. 2025 Oct 26;17(11):1389. doi: 10.3390/pharmaceutics17111389 (PMC12655201; doi:10.3390/pharmaceutics17111389)
Supplement: Supplementary file 1 [file pharmaceutics-17-01389-s001.zip › pharmaceutics-3858645-supplementary.pdf]

*pharmaceutics*

*Supplementary materials*

# The Influence of the External Chemistry of Silica-Based Mesoporous Nanocarriers on Organ Tropism and the Inhibition of Pulmonary Metastases

**Wenping Ye[1,†], Yakai Yan[1,†], Liuyi Chen[1], Zhongrui Yang[1], Guangya Xiang[1,2] and Yao Lu[1,*]**

1 School of Pharmacy, Tongji Medical College, Huazhong University of Science and Technology, Wuhan 430030, China.
2. NMPA Key Laboratory for Quality Research and Control of Drug Products, Wuhan Institute for Drug and Medical Device Control, Wuhan 430075, China.

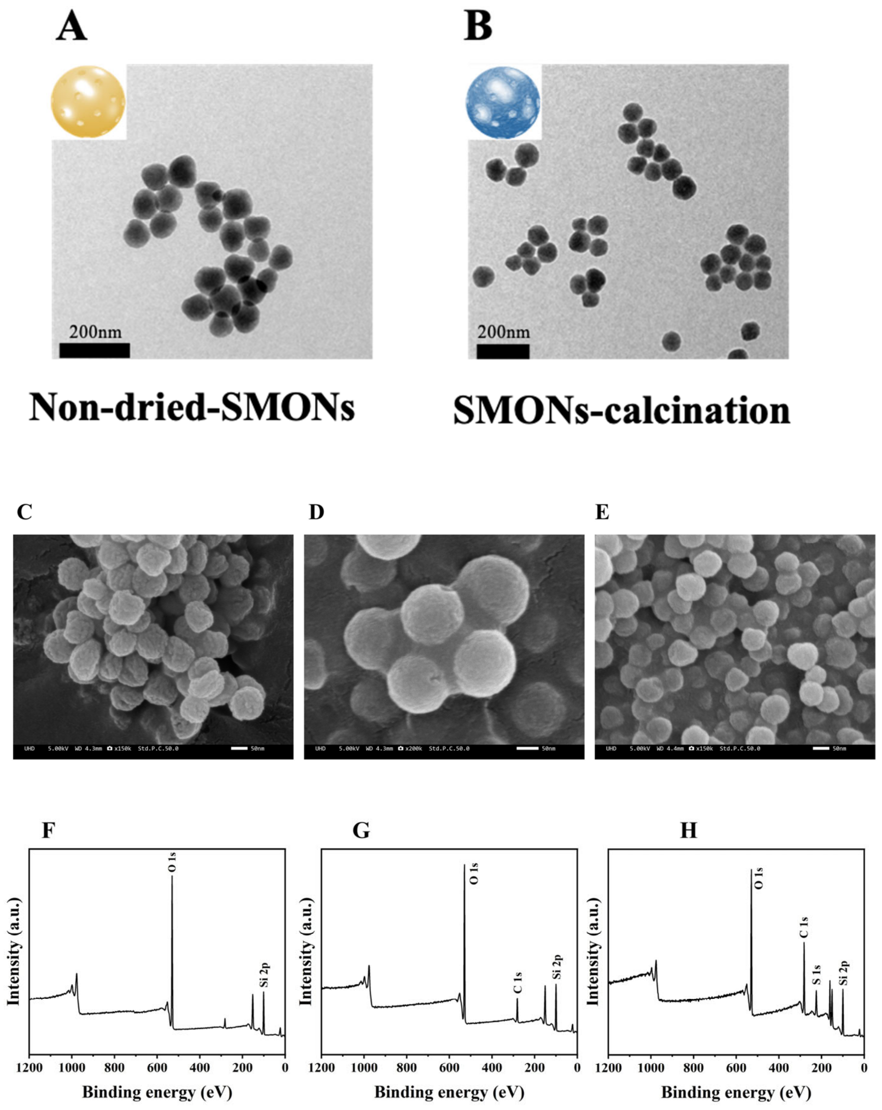


 https://doi.org/10.3390/pharmaceutics17111389

**Figure S1.** TEM images of MS NPs (**A, B**), SEM images of MSNs(**C**), CMONs(**D**), and HSMONs(**E**) and XPS analysis of MSNs(**F**), CMONs(**G**), and HSMONs(**H**).

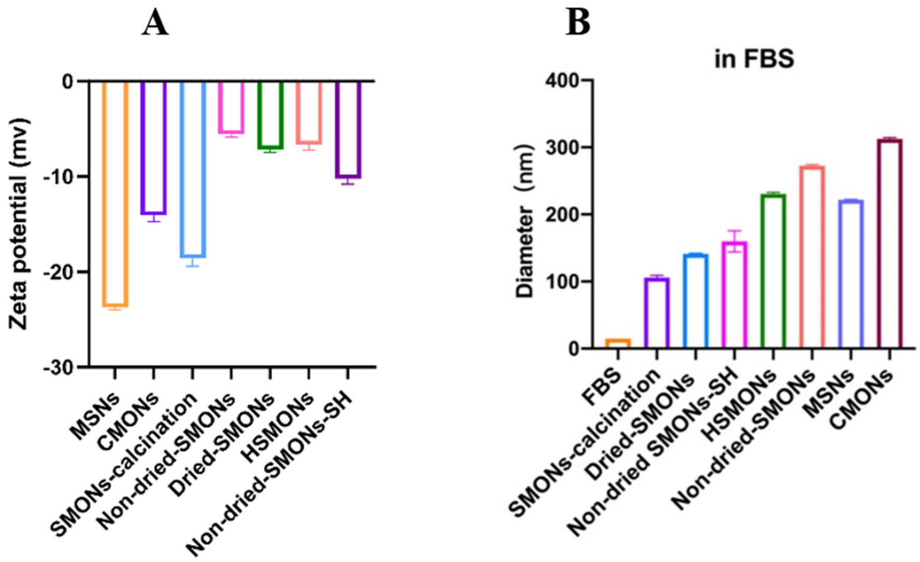


**Figure S2.** （**A**）Zeta potential of MS NPs and（**B**）particle size distribution of MS NPs in FBS（n = 3）.

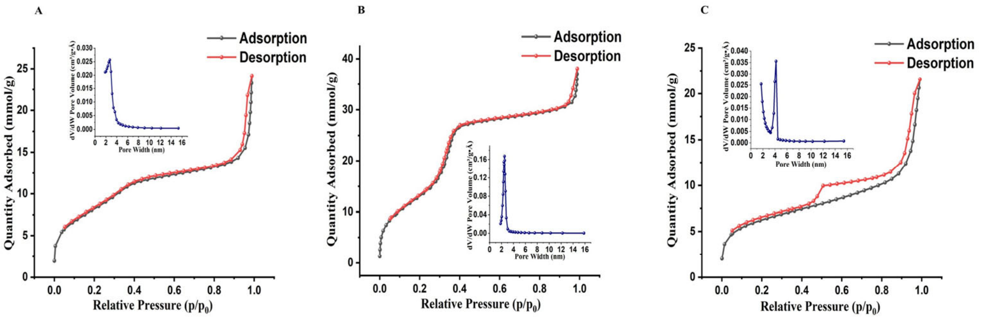


**Figure S3.** Nitrogen adsorption–desorption isotherms and pore size distribution curve of (**A**)MSNs, (**B**)CMONs, (**C**)HSMONs.

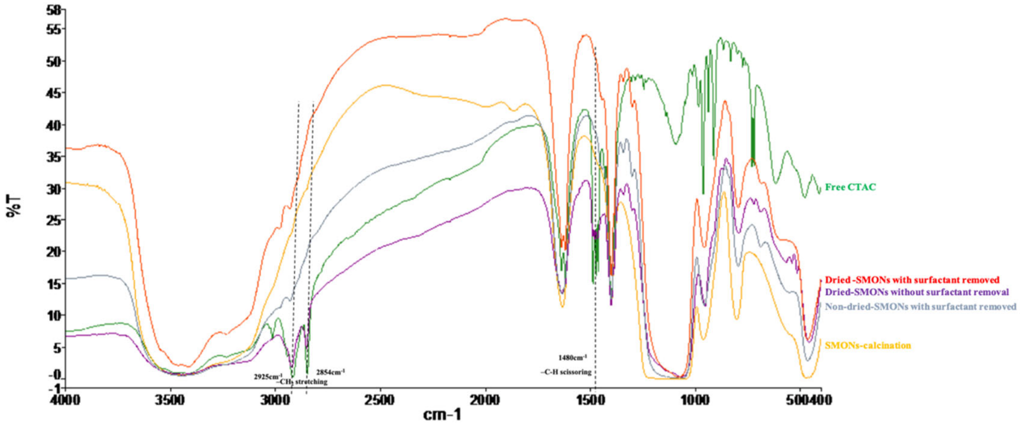

**Figure S4.** FTIR spectra of Free CTAC, Dried-SMNOs with surfactant removed, Dried-SMNOs without surfactant removal, Non-dried-SMNOs with surfactant removed, and SMONs-calcination.

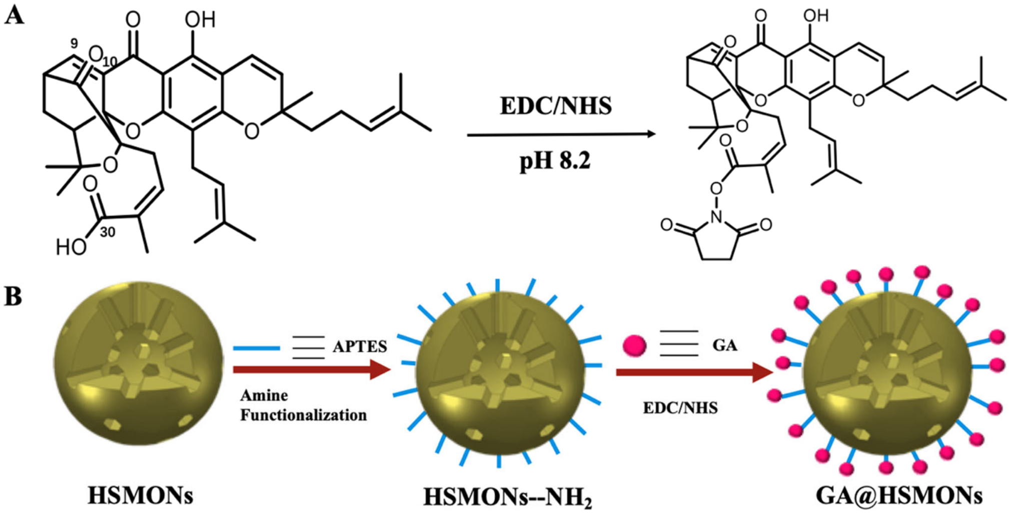


**Figure S5. (A)** Schematic diagram of GA carboxyl activation; **(B)** Surface modification and drug loading of MS NPs. In the schematic: red arrows denote the reaction procedure; blue lines stand for APTES; red dots stand for GA.

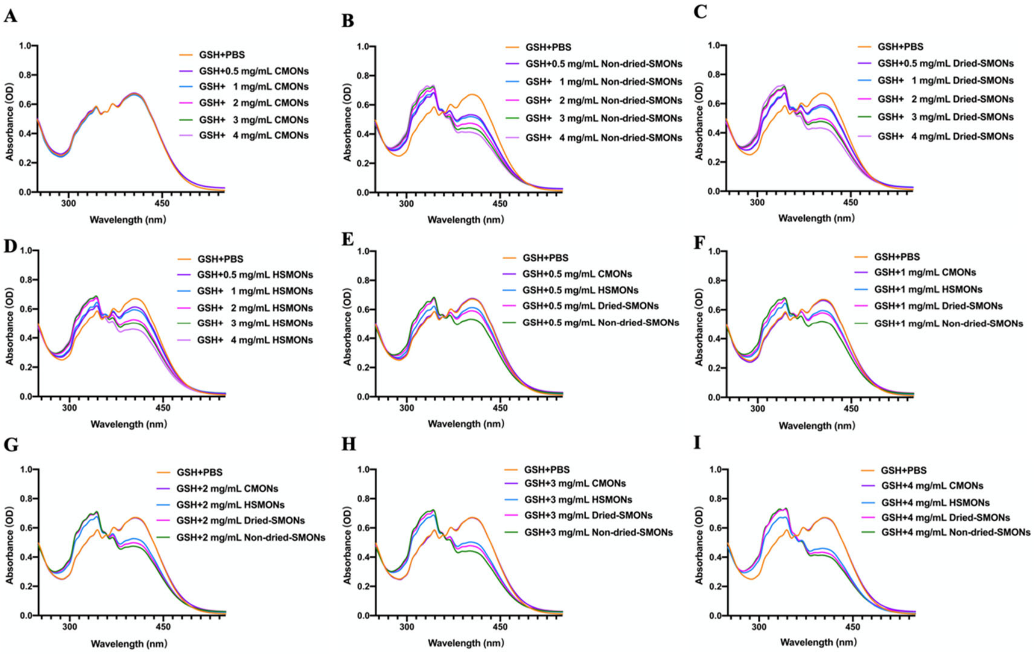


**Figure S6.** UV-Vis spectrum of DTNB for the detection of GSH contents. GSH content after reaction with different concentrations of MS NPs (**A, B, C, D**) and different NPs at the same concentration (**E, F, G, H, I**). The initial amount of GSH was 1 mL (0.8 mg/mL), and the amount of MS NPs was 1 mL (0.5, 1, 2, 3, 4 mg/mL).

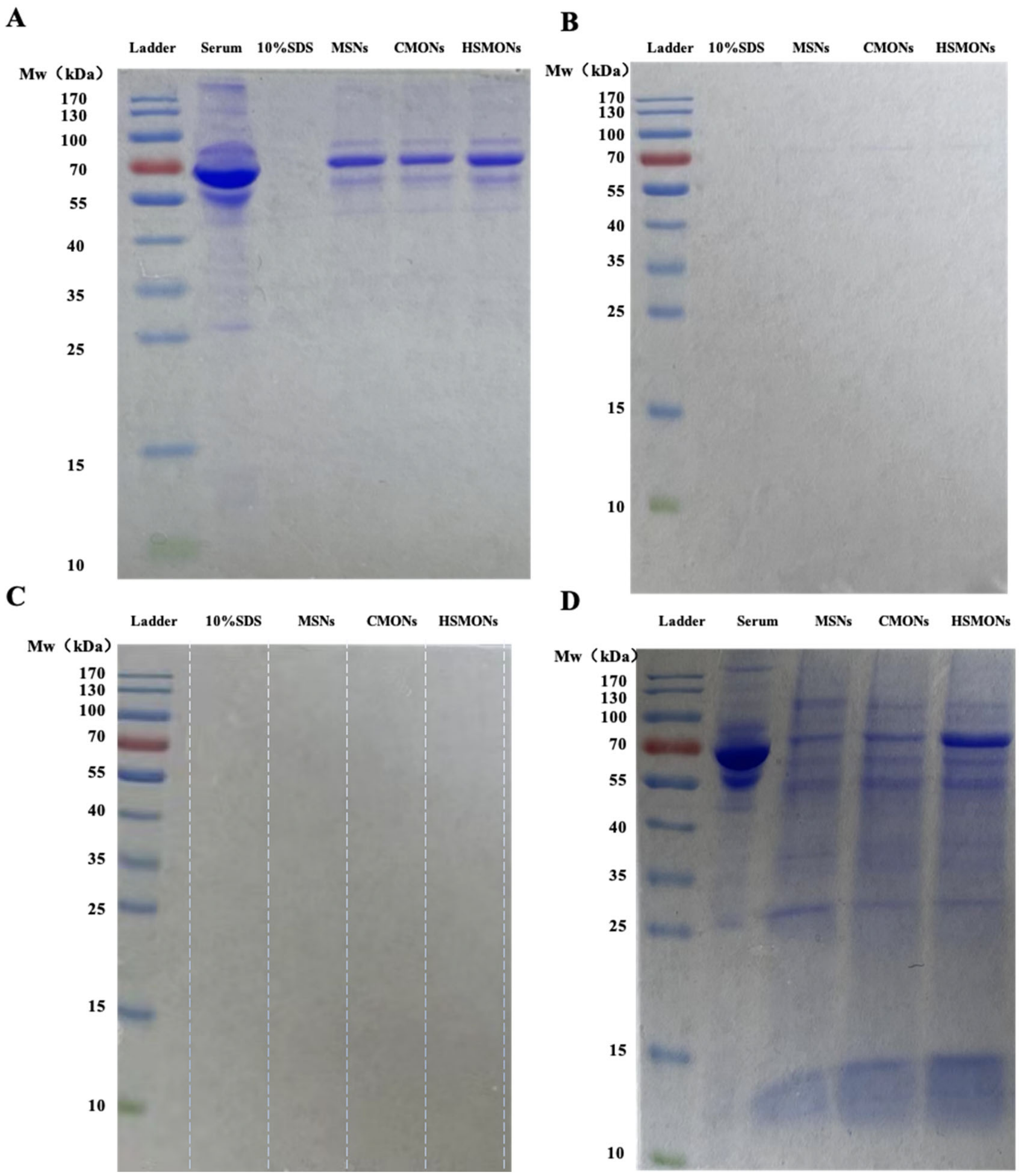


**Figure S7.** SDS-PAGE Analysis: (**A**) soft proteins of first wash from MS NPs with PBS in different groups; (**B**) second wash with PBS; (**C**) third wash with PBS; and (**D**) hard proteins after desorbed by 10% SDS from MS NPs.

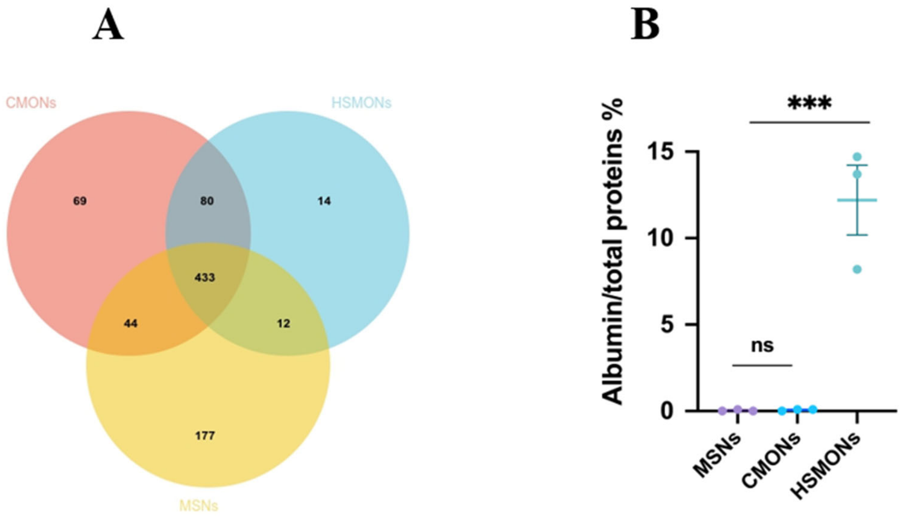


**Figure S8.** (**A**) The Venn diagrams for all identified proteins; (**B**)Albumin/total proteins in three groups were measured by LC-MS/MS. n.s. represented no significance; *** p <0.001.

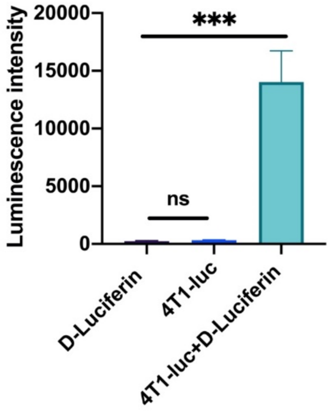


**Figure S9.** The microplate reader bioluminescence channel was used to assess the growth of 4T1-Luc cells and the bioluminescence signal of 4T1-Luc cells after the addition of D-luciferin potassium salt compared to free 4T1-luc cells and D-luciferin（n = 3）. n.s. represented no significance; *** p <0.001.

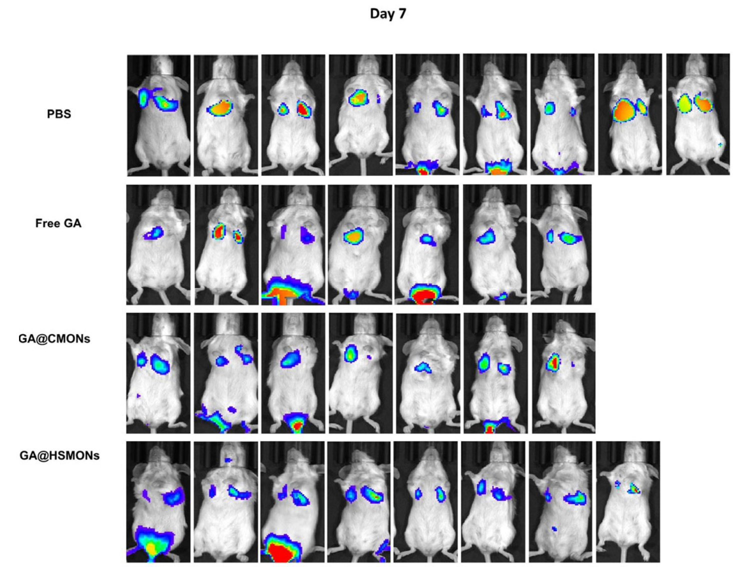
Day 7
PBS
Free GA
GA@CMONs
GA@HSMONs

**A**

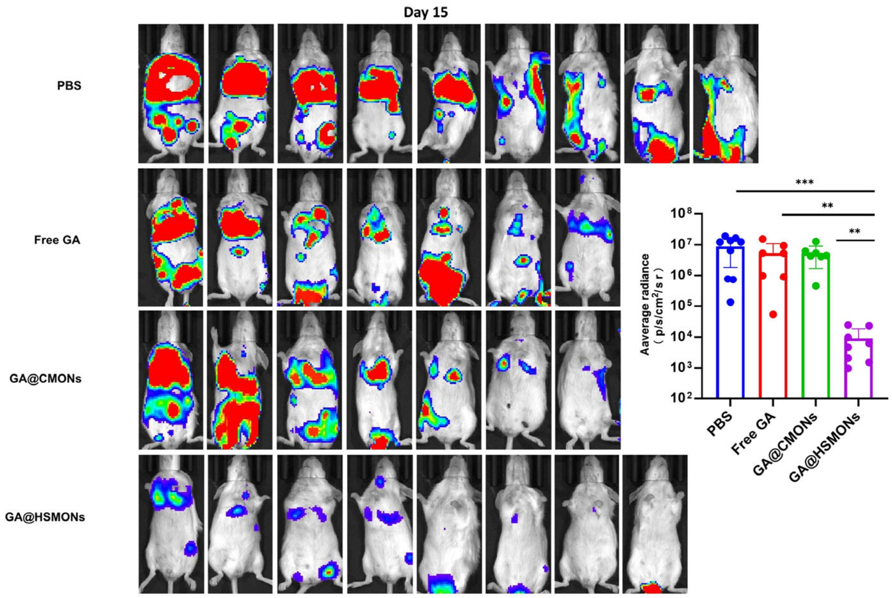
Day 15
PBS
Free GA
GA@CMONs
GA@HSMONs
***
**
**
Aaverage radiance
( p/s/cm²/s r )
10⁸
10⁷
10⁶
10⁵
10⁴
10³
10²
PBS
Free GA
GA@CMONs
GA@HSMONs

**B**

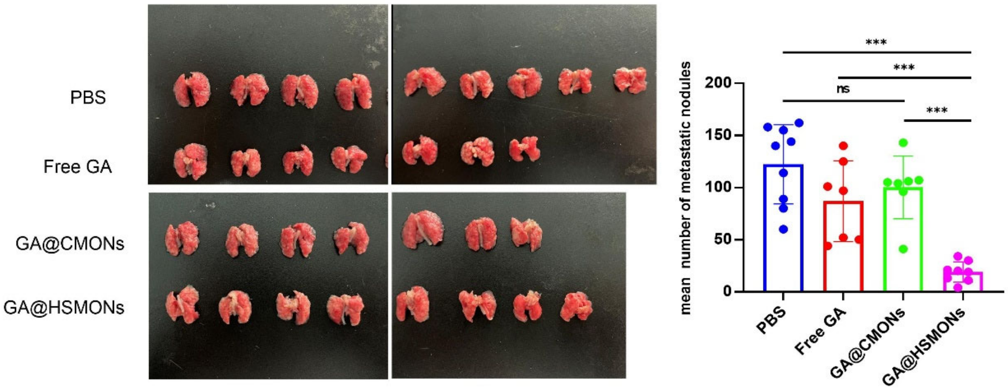


**C**

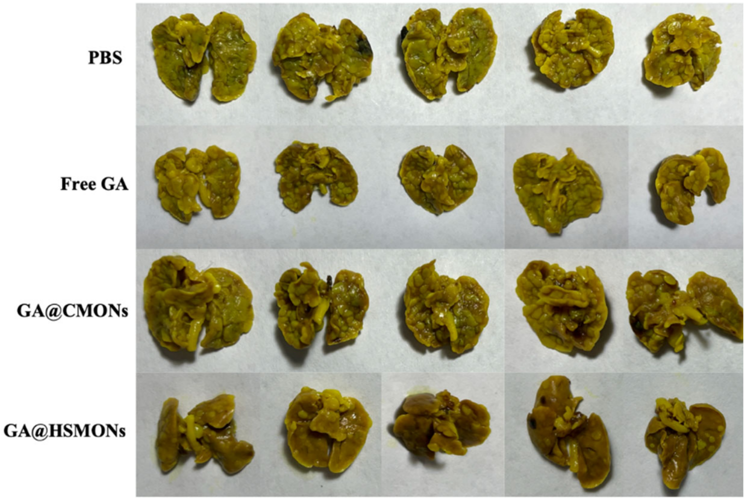


**D**

**Figure S10.** (**A**) Representative bioluminescence images of lung metastasis on Day 7; (**B**) Representative bioluminescence images of lung metastasis on Day 15; (**C**) Pictures of pulmonary nodules of 15-day lung tissue in different groups (4% paraformaldehyde fixation); (**D**) Pictures of pulmonary nodules of 15-day lung tissue in different groups (Bouin's fixative). *** p <0.001; ** p <0.01; * p <0.05, and n.s. represented no significance.

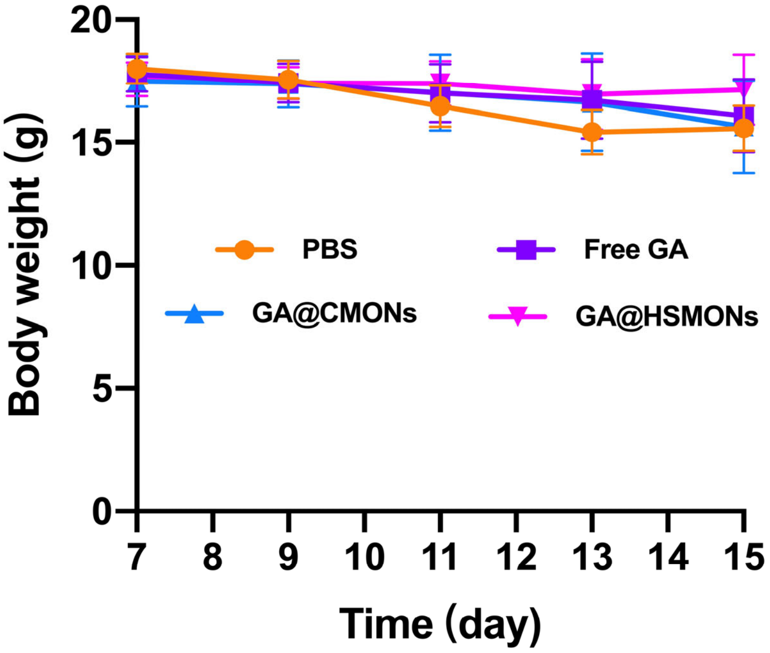


**Figure S11.** Changes in body weight of mice after different treatments (n = 5).

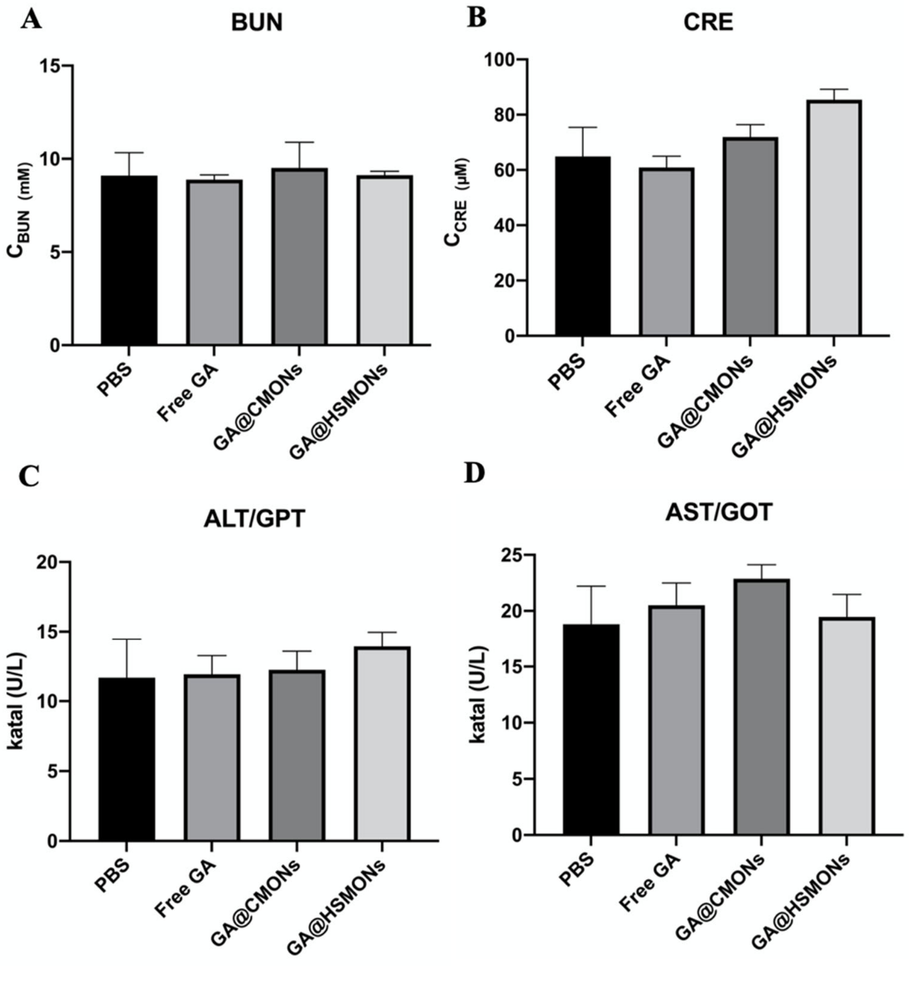

**Figure S12.** Effect of different treatments on serum (**A**) BUN, (**B**) CRE, (**C**) ALT/GPT and (**D**) AST/GOT levels.

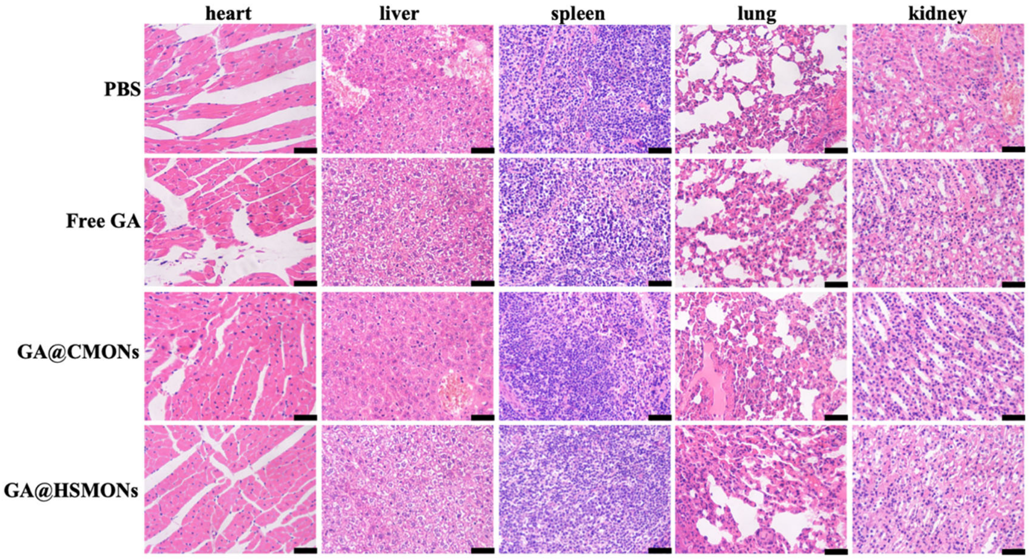


**Figure S13.** H&E-stained images of major organs of mice after various treatments, including heart, liver, spleen, lung and kidney (n = 3). Scale Bar = 20μm.

**Table S1.** Structural properties of MSNs, CMONs, and HSMONs.

| Sample name | $S_{BET}$ (m$^2$ g$^{-1}$) | Pore size (nm) |
|---|---|---|
| MSNs | 666.5 | 2.7 |
| CMONs | 1123.2 | 2.5 |
| HSMONs | 483.9 | 4.2 |

**Table S2.** Characterization of GA@NPs.

| | Coupling efficiency（%） | Drug loading efficiency（%） |
|---|---|---|
| GA@HSMONs | 36.98% ± 3.94% | 10.06% ± 0.08% |
| GA@CMONs | 29.86% ± 1.25% | 7.54% ± 0.02% |
